# Patient satisfaction after unilateral oncoplastic volume displacement surgery for breast cancer, evaluated with the BREAST-Q™

**DOI:** 10.1186/s12957-019-1640-6

**Published:** 2019-06-05

**Authors:** Anna Gardfjell, Cecilia Dahlbäck, Kristina Åhsberg

**Affiliations:** 1 Department of Surgery, Region Blekinge Hospital, Karlskrona, Sweden; 2 Department of Clinical Sciences Lund, Surgery, Skåne University Hospital, Lund University, Lund, Sweden

## Abstract

**Background:**

Oncoplastic breast-conserving surgery allows larger resections in unfavorable locations, with an improved chance of preserving esthetics. Indications and timing for potential contralateral surgery to obtain symmetry are not clear. The aim of this study was to evaluate patient satisfaction after unilateral oncoplastic volume displacement surgery, to investigate potential risk factors for lower patient satisfaction and to assess patient wish for contralateral surgery.

**Method:**

A cohort of 144 women, consecutively treated for breast cancer with unilateral breast-conserving oncoplastic volume displacement surgery, followed by radiotherapy and with an unoperated contralateral breast, was sent the BREAST-Q™ breast-conserving therapy (BCT) and a study-specific questionnaire. In all, 120 women (83%) responded. For these women, the median value for resected specimen weight was 92 g (range 14–345) and for the estimated percentage of the breast volume excised 15% (range 3–35%).

**Results:**

The median patient-reported score for “Satisfaction with breast” (BREAST-Q™ BCT) was 74/100. Factors associated with a score below median value in a simple logistic regression model adjusted for age and BMI were axillary clearance (OR 2.46, 95% CI 1.09–5.56), neoadjuvant chemotherapy (OR 3.26, 95% CI 1.15–9.24), and low breast density (OR 2.32 95% CI 1.02–5.29). Thirteen women (11%) were interested in contralateral surgery.

**Conclusion:**

Most patients in this study cohort, who had undergone breast-conserving therapy with oncoplastic volume displacement techniques, were satisfied with their breasts without surgery to the contralateral breast. This indicates that contralateral surgery to achieve symmetry only should be performed after individual evaluation and as a delayed procedure.

## Background

Breast-conserving therapy (BCT) is the preferred treatment for early-stage breast cancer [1], with equivalent survival [2, 3], and esthetic, functional, and psychosocial advantages compared to mastectomy [4, 5]. Ideally, the breast should appear normal-looking after BCT. However, studies have shown that up to 30% of patients have reported unsatisfactory esthetic results that can demand surgical revision [6]. Large excision volume is a well-studied risk factor for a poor esthetic outcome [7–12], the risk increasing if the resection exceeds 15–20% of the breast volume [7, 8, 12]. In the medial part of the breast, less of the breast volume can be resected with preserved esthetics (5%) [9]. Beyond possible asymmetry, deformity can also arise due to tissue necrosis, especially in fatty breasts, caused by large dual-plane mobilizations to cover the resected portion [13]. Tumor location in the medial, inferior, or central parts of the breast is also related to lower satisfaction after BCT [10, 11, 14, 15]. In addition, postoperative radiotherapy (PRT) can accentuate a suboptimal esthetic result by substantial and unpredictable shrinkage [16–18]. Other observed risk factors for lower patient satisfaction after BCT have been high body mass index (BMI) [12, 14], axillary clearance [12], postoperative complications [11, 12, 14], reoperative procedures [10, 12, 14], adjuvant chemotherapy [15], large scars after resection [10], and how the patient perceived the preoperative information and possibility to participate in decision-making regarding surgical technique [19]. The impact of these factors has varied between studies.

Where BCT is contraindicated due to poor expected esthetic outcome, the use of oncoplastic breast-conserving surgery (OPS) has rapidly increased during the last decade [20]. OPS can allow a wider excision of the tumor, thereby ensuring safer margins and widening the possibility of breast-conserving surgery. For some women, this can spare them a mastectomy, without compromising local control. In addition, a so called oncoplastic mammoplasty (OPS with techniques similar to a reduction mammoplasty) can be performed if the patient has large breasts, and a reduction of the breast volume is beneficial to simplify PRT. After operation with a volume displacement technique, a slight asymmetry can be expected regarding size and ptosis of the breast, although the shape of the breast and centralization of the nipple is preserved [13]. As symmetry is presented to be of great importance for patient satisfaction [14, 17, 21], many breast centers also operate on the contralateral breast in the primary setting to ensure postoperative symmetry. However, there are no firm guidelines specifying in which cases contralateral surgery should be performed, and a recent interview study showed that patients accept a slight asymmetry due to concerns regarding risks with surgery and the will to preserve the untouched contralateral, healthy breast [22].

Patient-reported outcome measures (PROMs), are essential instruments to gain information regarding the patient’s experience and health-related quality of life (HR-QoL). The BREAST-Q™ questionnaires are currently the only validated, disease-specific questionnaires, designed to evaluate different aspects of HR-QoL and patient satisfaction after breast cancer surgery [23]. The BREAST-Q™ modules are used globally, and have been used in studies to compare various surgical techniques for breast cancer [24]. To date, there are five studies that have used the BREAST-Q™ BCT to evaluate results after BCT [25–29]. Although some of the studies have included patients operated with various oncoplastic techniques, no study has yet evaluated a cohort of patients consistently operated with volume displacement OPS with the BREAST-Q™ BCT.

## Method

### Study aim

The aim of this study was to evaluate postoperative patient satisfaction, using the BREAST-Q™ BCT, after unilateral volume displacement OPS and radiotherapy, without surgery to the contralateral breast. Secondary aims were to find potential determinants for poor patient satisfaction and to assess patient wish to undergo contralateral surgery.

### Participant recruitment

**Fig. 1:**
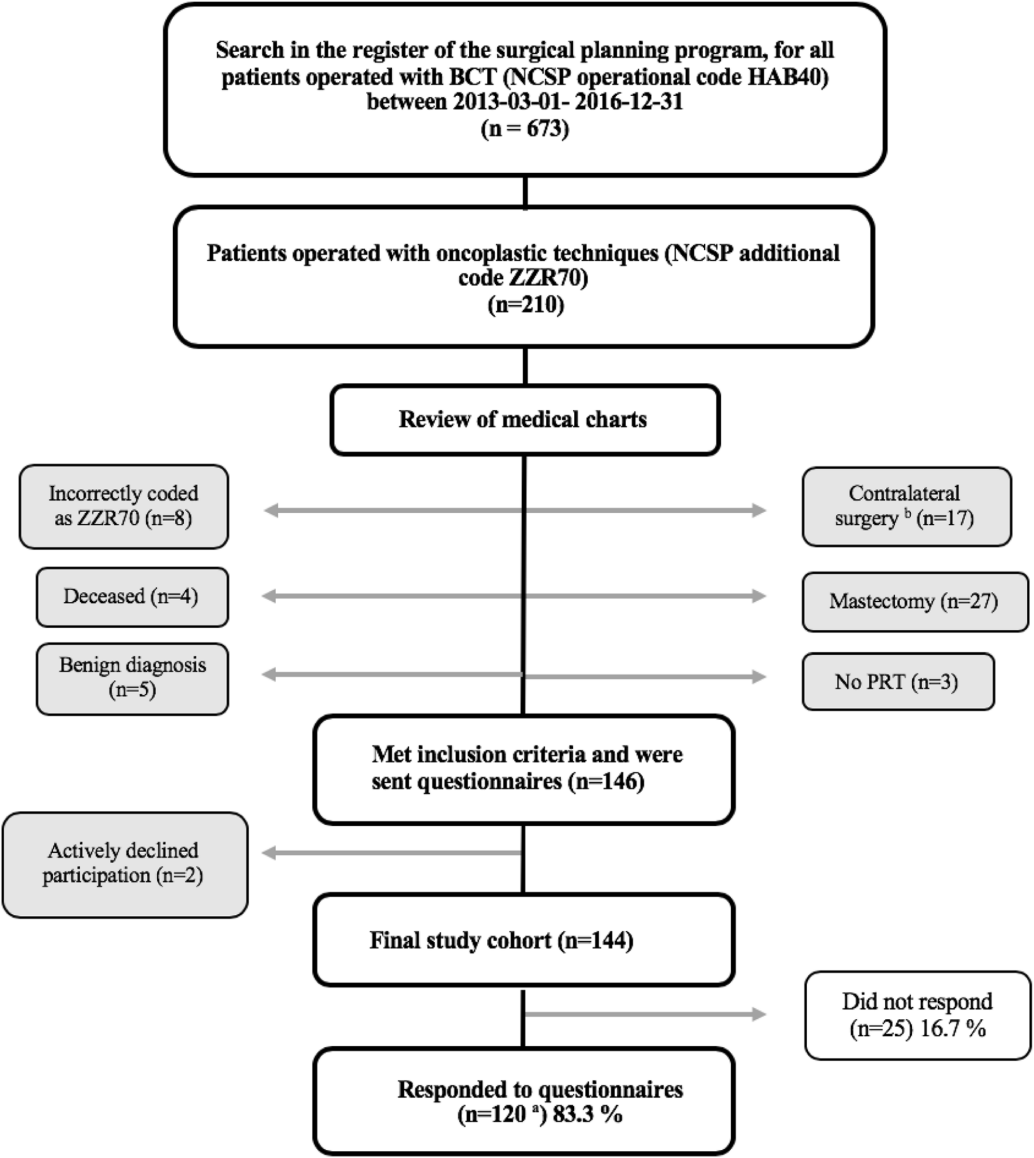
Flow chart of the study recruitment. Grey boxes represent patients not included in the study cohort. ^a^One patient responded only to the study-specific questionnaire. ^b^Contralateral surgery was performed due to bilateral cancer (*n* = 3), prior breast cancer (*n* = 8), to achieve symmetry (*n* = 6). PRT postoperative radiotherapy, NCSP Nordic Medico-Statistical Committee (NOMESCO) classification of surgical procedures, HAB40 “wedge excision of mammary gland”, ZZR70 “flap of glandular tissue”

Women consecutively operated with unilateral oncoplastic breast-conserving surgery for malignant breast disease between 1 March 2013 and 31 December 2016 at the Skåne University Hospital in Lund were identified through a systematical search in the local surgery planning program. All patients coded with Nordic Medico-Statistical Committee (NOMESCO) classification of surgical procedures (NCSP [30]), operational code HAB40 (wedge excision of the mammary gland), were located (*n* = 673). Of these, 210 patients had the additional code of ZZR70 (flap of mammary gland tissue) and represented the number of women recorded as operated with volume displacement oncoplastic surgery (Fig. 1).

A review of medical charts was performed prior to study inclusion to ascertain that PRT had been completed at least 1 year prior to the study and that no surgery to the contralateral breast had been performed (Fig. 1). In total, 64 women were excluded from the study. Eight women had been incorrectly coded with ZZR70. Five women had a benign diagnosis. Twenty-seven women had been reoperated with a mastectomy: twenty-five due to positive surgical margins, one due to a local recurrence, and one had undergone prophylactic bilateral mastectomy due to a BRCA-1 mutation. Three women had not received PRT: one due to a preexisting heart condition, one declined treatment, and for one patient the histopathological analysis showed malignant phyllodes tumor, for which PRT is not indicated. Seventeen women had undergone bilateral surgery: eight women had been operated for a previous cancer, three women had bilateral cancers, and six women were operated on the contralateral breast to achieve symmetry. Four women had died prior to the study: three from metastatic breast cancer and one from a stroke. The remaining 146 women were sent the questionnaires*.* Two patients declined participation and were excluded, and all collected data from their medical charts were deleted.

### Data collection

Data were collected regarding OPS technique, tumor size and location, postoperative complications, reoperations due to inadequate margins, pathological anatomical diagnosis, volume and weight of the specimen, tobacco use, patient age and BMI at time of surgery, and neoadjuvant and adjuvant treatment. Postoperative complications were defined by the Clavien-Dindo classification system [31]. Only complications that concerned the breast were included. Mammograms were assessed to estimate preoperative breast tissue composition and calculate breast volume. The algorithm for volume calculation uses measurements of width, *w*, and height, *h*, from the craniocaudal projection of the mammogram together with the measurement of compression, *c* (thickness of the breast when compressed during mammography), defining the breast as a half-elliptical cylinder; $$ \mathrm{volume}\ \left({\mathrm{cm}}^3\right)=\raisebox{1ex}{$\pi $}\!\left/ \!\raisebox{-1ex}{$4$}\right.\times whc $$ (cm)*.* The method has been validated by Kalbhen et al. [32] and Kayar et al. [33]. Preoperative composition of breast tissue was evaluated by a senior consultant of radiology, who categorized the images into groups A–D, in accordance with the American College of Radiology’s Breast Imaging Reporting and Data System (BI-RADS®) 5th edition [34]. Specimens were weighed to the closest gram at the surgical theater directly after excision, before formalin fixation. Fresh specimen weight was used as a surrogate for volume in the current study, assuming that breast tissue weighs 1 g/cm^3^, which has been implemented in previous studies [8, 12]. Estimated percentage of breast volume excised (EPBVE) was calculated by dividing specimen weight with preoperative breast volume. Follow-up time was defined as the number of months between the date of operation and the date when the questionnaires were sent out to the individual.

### Surgery and systemic treatment

OPS is used at our unit if the tumor is located caudally, medially, or centrally, or where tumor/breast volume ratio is > 20%. It is also an option at reoperation after an initial wide local excision (WLE) with positive margins. Most of the performed techniques are those presented by Clough et al. in Ann Surg Oncology 2010 [13], and most cases fall under the volume displacement category of OPS. At this kind of operation, routinely, only the cancer-burdened breast is operated in the primary setting unless a reduction mammoplasty is favorable to simplify PRT.

Preoperatively, radiological tumor location and extent was assessed with full-field digital mammography and ultrasonography. Before neoadjuvant treatment, a clip was placed in the tumor. At the preoperative multidisciplinary conference, a recommendation is made for mastectomy or breast-conserving surgery for the individual patient, but the definitive choice of breast-conserving technique and use of oncoplastic procedures is made by the surgeon who sees the patient. For non-palpable lesions, a wire was placed in the tumor or area of calcifications before surgery. Operations were performed by seven breast surgeons with training in OPS. Two consultants performed a majority of the operations, 43.1% (*n* = 62) and 25.7% (*n* = 37), respectively, with the remaining five surgeons performing 31.2% (*n* = 45). Patient satisfaction in relation to the surgeon was not analyzed. Sentinel node biopsy with dual technique was routinely used to assess axillary node status. Axillary clearance was performed in patients with positive lymph nodes, if not included in the SENOMIC trial [35] or randomized to no further surgery in the SENOMAC trial [36]. If the placement of the breast incision is permitted, the same incision was used for axillary surgery. Otherwise, a separate incision was made. An intraoperative X-ray of the resected specimen was performed to ensure a radiological margin of minimum 10 mm. If radiological margins were inadequate, additional tissue was excised during the same operation. Pathological evaluation of the margins by frozen section is not used routinely at our unit. Patients were treated neoadjuvantly and adjuvantly in accordance with the Swedish national guidelines for breast cancer treatment [37], involving radio-, endocrine, chemo-, and anti-HER2 therapy, depending on tumor stage and histopathological profile.

### Questionnaires

The BREAST-Q™ BCT is a standardized, validated disease-specific PROM developed to evaluate patient satisfaction and HR-QoL after BCT. It is divided into nine domains for evaluation of satisfaction with breasts; adverse effects of radiation; psychosocial, physical and sexual well-being; perception of information prior to surgery; and interactions with different categories of medical staff. [23]. Patients were provided with a linguistically validated Swedish translation of the questionnaire [26].

Some questions of special interest to the current study were not included in the BREAST-Q™ BCT. Therefore, a study-specific questionnaire was constructed which included questions assessing satisfaction regarding different aspects of the operated breast, satisfaction with preoperative information, perception of ability to participate in the preoperative decision-making process, and wish for corrective surgery of either breast. Inspiration was taken from a previous study from SUS Malmö Surgical Center [26] that evaluated patient satisfaction after conventional breast-conserving surgery (BCS).

### Statistical methods and data analysis

The BREAST-Q™ BCT was analyzed as complete domains with a manual scoring table provided by the developers (http://qportfolio.org/breast-q/breast-cancer/). Raw data were thus converted to a “*Q*-score” ranging from 0 to 100. A high score is meant to correspond to a higher HR-QoL. Patients who answered less than 50% of the questions on a scale were excluded from that specific scale. Missing values up to 50% of the scale were replaced by calculating the mean value of the answered questions and using this as a surrogate for the missing value(s), thus completing all items of the scale.

Descriptive statistics for parametric variables were expressed as median, interquartile range (IQR), and range due to a skewed distribution in the continuous variables. Descriptive statistics for non-parametric variables were presented as frequencies and percentages. Fisher’s exact test was used to find differences between subgroups (dichotomous variables). Spearman’s rho was used to analyze correlations between different domains of the BREAST-Q™ (continuous *Q*-scores) and between the BREAST-Q™ (continuous *Q*-scores*)* and the study-specific questionnaire (4-point scales). The associations between *Q*-scores and potential determinants for lower satisfaction were analyzed by dichotomizing *Q*-scores with a cutoff at the median value. Logistic regression analysis was used to investigate the associations. Odds ratios (OR) with 95% confidence intervals (CI) were obtained. Associations with potential risk factors for lower satisfaction after unilateral volume displacement OPS were first analyzed in a simple logistic regression model, and thereafter in a model adjusted for BMI and age, and finally in a multiple logistic regression model including factors in which a statistically significant association had been observed in the simple logistic regression analysis.

Statistical analyses were performed using IBM® SPSS Statistics for Macintosh, Version 24.0. IBM Corp., Armonk, NY.

## Results

### Demographics of responding patients

Response rate was 83.3%, as 120 of the included 144 women responded (Fig. 1). One patient answered only the study-specific questionnaire, and the remaining 119 answered both questionnaires. Patient, tumor, and treatment characteristics for the total cohort and for responders and non-responders, respectively, are shown in Table 1. For responding patients, median age at operation was 61 (33–80) years and median BMI 25.2 (16.0–42.0) kg/m^2^, median follow-up time 28 (15–54) months, median radiological and histological tumor extents (including malignant microcalcifications and DCIS) were 20 (5–60) mm and 22 (6–90) mm, respectively, specimen weight 92 (14–345) grams and EPBVE 15% (3–35%).

**Table 1:** Patient, tumor, and therapy characteristics. All values are presented as median (range) or percentage (count) of subgroup

	Total cohort (*n* = 144)	Responders (*n* = 120)	Non-responders (*n* = 24)
Age (years)	60 (32–80)	61 (33–80)	52 (32–79)
BMI (kg/m^2^)	25.1 (16.0–42.0)^d^	25.2 (16.0–42.0)	24.2 (19.7–34.6)
Smoker	14.0% (*n* = 20)	11.8% (*n* = 14)	25.0% (*n* = 6)
Radiological tumor extent (mm)	20 (5–60)	20 (5–60)	25 (6–60)
Histological tumor extent (mm)	23 (6–90)	22 (6–90)	25 (10–51)
Specimen weight (g)	86 (14–345)^e^	92 (14–345)	62 (33–161)
Breast volume (cm^3^)^a^	644 (110–2033)^f^	644 (110–2033)	637 (200–1395)
EPBVE (%)^b^	14 (3–35)^g^	15 (3–35)	13 (7–28)
Oncoplastic method			
Raquet	49.3% (*n* = 71)	54.2% (*n* = 65)	25.0% (*n* = 6)
Round block	21.5% (*n* = 31)	20.0% (*n* = 24)	29.2% (*n* = 7)
V-mammoplasty	13.2% (*n* = 19)	10.8% (*n* = 13)	25.0% (*n* = 6)
Inverted-T	7.6% (*n* = 11)	7.5% (*n* = 9)	8.3% (*n* = 2)
J-mammoplasty	6.9% (*n* = 10)	6.7% (*n* = 8)	8.3% (*n* = 2)
Other	1.4% (*n* = 2)	0.8% (*n* = 1)	4.2% (*n* = 1)
Axillary clearance	27.8% (*n* = 40)	30.0% (*n* = 36)	16.7% (*n* = 4)
Re-excision	4.9% (*n* = 7)	4.2% (*n* = 5)	8.3% (*n* = 2)
Postoperative complications of the breast^c^			
Grade 1	2.1% (*n* = 3)	2.5% (*n* = 3)	0
Grade 2	2.1% (*n* = 3)	0.8% (*n* = 1)	8.3% (*n* = 2)
Grade 3	1.4% (*n* = 2)	1.7% (*n* = 2)	0
Neoadjuvant chemotherapy	17.4% (*n* = 25)	17.5% (*n* = 21)	16.7% (*n* = 4)
Adjuvant chemotherapy	25.0% (*n* = 36)	26.7% (*n* = 32)	16.7% (*n* = 4)
Adjuvant endocrine therapy	72.2% (*n* = 104)	74.2% (*n* = 89)	62.5% (*n* = 15)
Postoperative radiotherapy	100% (*n* = 144)	100% (*n* = 120)	100% (*n* = 24)
Breast density			
High (BI-RADS® C–D)	30.6% (*n* = 55)	54.2% (*n* = 13)	35.0% (*n* = 42)
Low (BI-RADS® A–B)	62.5% (*n* = 89)	45.8% (*n* = 11)	65.0% (*n* = 78)
Follow-up time (months)	27 (15–54)	28 (15–54)	24 (15–54)

*BMI* body mass index, *EPBVE* estimated percentage of breast volume excised, *BI-RADS****®*** Breast Imaging Reporting and Data System

^a^Breast volume calculated from mammograms by height × width × compression (cm) × *π*/4

^b^EPBVE calculated by dividing specimen weight with breast volume

^c^Complications defined by the Clavien-Dindo classification [31]

^d^One missing value in the “non-responder” group

^e^Eight missing values, out of which seven were in the “responder” group and one in the “non-responder”

^f^Two missing values in the “responder” group

^g^Ten missing values, out of which nine were in the “responder” group and one in the “non-responder” group

### Surgical and treatment specifications of responding patients

The most frequent technique, the Raquet oncoplasty, was used in 54.2% of the patients (*n* = 65). Axillary clearance was performed in 25.0% (*n* = 30). Two women were reoperated with OPS after an initial WLE with insufficient margins. Five women (4.2%) were re-excised after the initial OPS with insufficient margins, with a final result of BCT. Neoadjuvant chemotherapy was administered to 17.5% (*n* = 21). Adjuvant chemo- and endocrine therapy were given to 26.7% (*n* = 32) and 74.2% (*n* = 89), respectively. All women received PRT.

### Complications of responding patients

Six women (5.0%) had complications from the breast area (Table 1): three grade 1 complications (one hematoma and two minor skin necroses), one grade 2 complication (infection treated with antibiotics), and two grade 3b complications (reoperation due to postoperative bleeding). Due to infection, adjuvant radiotherapy was delayed by three weeks in one case.

### Patient satisfaction

The median *Q-*scores of the BREAST-Q™ BCT domains were for “Satisfaction with breast” 74/100 (IQR 59–85), “Psychosocial well-being” 87/100 (IQR 57–100), and “Sexual well-being” 60/100 (IQR 40–69). Of the women, 97.5–100% answered more than half of the BREAST-Q™ BCT domains, with an exception for “Sexual QoL”, in which the completion percentage was 64.7% (Table 2).

**Table 2:** *Q*-scores for BREAST-Q™ BCT Domains

Domain	*n*	Median	IQR	Range	Mean (SD)
1. Satisfaction with breasts	119	74	59–85	12–100	72 (20)
2. Adverse effects of radiation	118	100	89–100	49–100	94 (11)
3. Psychosocial well-being	119	87	57–100	14–100	78 (23)
4. Sexual well-being	77	60	40–69	0–100	55 (21)
5. Physical well-being	119	78	69–92	21–100	78 (18)
6. Satisfaction with information from breast surgeon	119	75	58–100	37–100	76 (19)
7. Satisfaction with breast surgeon	118	100	92–100	23–100	92 (14)
8. Satisfaction with medical team	118	100	100–100	31–100	96 (12)
9. Satisfaction with office staff	116	100	100–100	44–100	97 (9)

Number of responders and descriptive statistics of Q-scores from domains of the BREAST-Q™ BCT

*IQR* interquartile range, *SD* standard deviation, *n* number of responders (maximum 119 patients since one of the 120 responding patients only answered the study-specific questionnaire)

In the study-specific questionnaire, 88.3% (*n* = 106) of the women were satisfied or very satisfied with the appearance of the operated breast, 86.7% (*n* = 104) with the size of the operated breast, 88.3% (*n* = 106) with the shape of the operated breast, 78.3% (*n* = 94) with the symmetry between the operated and untouched breast, 90.8% (*n* = 109) with the nipple-areolar complex (NAC) positioning, 85.8% (*n* = 103) with the appearance of the NAC, 83.3% (*n* = 100) with the scar, and 79.2% (*n* = 95) with skin sensitivity (Table 3).

**Table 3:** Descriptive statistics of answers to the study-specific questionnaire

Expectations, information, participation in decision-making					
	Not at all	Partly	Almost entirely	Entirely	Missing
Has the operation met your expectations regarding the cosmetic outcome?	1 (0.8%)	8 (6.7%)	30 (25.0%)	79 (65.8%)	2 (1.7%)
Did you receive enough information about the expected cosmetic outcome?	6 (5.0%)	14 (11.7%)	24 (20.0%)	74 (61.7%)	2 (1.7%)
Did you perceive an opportunity to participate in the decision making process?	20 (16.7%)	16 (13.3%)	20 (16.7%)	62 (51.7%)	2 (1.7%)
Satisfaction with different aspects of the operated breast					
	Dissatisfied	Not entirely satisfied	Satisfied	Very satisfied	Missing
Appearance of the operated breast	4 (3.3%)	10 (8.3%)	42 (35.0%)	64 (53.3%)	0
Size of the operated breast	4 (3.3%)	11 (9.2%)	46 (38.3%)	58 (48.3%)	1 (0.8%)
Shape of the operated breast	3 (2.5%)	10 (8.3%)	47 (39.2%)	59 (49.2%)	1 (0.8%)
NAC positioning	2 (1.7%)	8 (6.7%)	44 (36.7%)	65 (54.2%)	1 (0.8%)
NAC appearance	2 (1.7%)	12 (10.0%)	46 (38.3%)	57 (47.5%)	3 (2.5%)
Symmetry	6 (5.0%)	19 (15.8%)	50 (41.7%)	44 (36.7%)	1 (0.8%)
Appearance of scar	4 (3.3%)	13 (10.8%)	47 (39.2%)	53 (44.2%)	3 (2.5%)
Skin sensitivity	5 (4.2%)	18 (15.0%)	56 (46.7%)	39 (32.5%)	2 (1.7%)
Wish regarding additional/corrective/other surgery					
	No		Yes	Missing	
Would you rather have removed the entire breast?	114 (95.0%)		3 (2.5%)	3 (2.5%)	
Would you like to have corrective surgery of the operated breast?	109 (90.8%)		8 (6.7%)	3 (2.5%)	
Would you like to have a procedure of the contralateral breast for symmetry?	101 (84.2%)		13 (10.8%)	6 (5.0%)	

*NAC* nipple areolar complex

Scores for the study-specific questionnaire, presented as count and percentage of responding patients in brackets

Determinants for patient satisfaction in the BREAST-Q™ BCT domain “Satisfaction with breast” below median value (*Q*-score < 74) in the crude logistic regression analysis were found to be specimen weight > 100 g, axillary clearance, neoadjuvant therapy, and breasts with low density (BI-RADS® A-B). The factors that remained significant when adjusted for age and BMI were axillary clearance (OR 2.46, 95% CI 1.09–5.56), neoadjuvant chemotherapy (OR 3.26, 95% CI 1.15–9.24), and low breast density (OR 2.32, 95% CI 1.02–5.29) (Table 4). No independent risk factors were found in the multiple logistic regression model (Table 5).

**Table 4:** Logistic regression model of potential risk factors for lower patient satisfaction

Factor	*Q*-score ≥ 74	*Q*-score < 74	OR (95% CI)^a^	OR (95% CI)^b^
BMI (kg/m^2^)				
< 25	31 (54.4%)	26 (45.6%)	1	1
≥ 25 to < 30	21 (47.7%)	23 (52.3%)	1.31 (0.59–2.87)	1.28 (0.58–2.84)
≥ 30	9 (50.0%)	9 (50.0%)	1.19 (0.41–3.44)	1.18 (0.40–3.46)
Age (years)				
< 50	18 (56.3%)	14 (43.8%)	1	1
50–65	21 (46.7.7%)	24 (53.3%)	1.47 (0.59–3.66)	1.44 (0.58–3.60)
> 65	22 (52.4%)	20 (47.6%)	1.17 (0.46–2.95)	1.14 (0.45–2.91)
BI-RADS® classification of breast density				
High density(C–D)	27 (64.3%)	15 (35.7%)	1	1
Low density (A–B)	34 (44.2%)	43 (55.8%)	*2.28 (1.05–4.94)*	*2.32 (1.02–5.29)*
Smoker				
No	53 (52.5%)	48 (47.5%)	1	1
Yes	4 (28.6%)	10 (71.4%)	2.76 (0.81–9.38)	2.59 (0.74–9.12)
EPBVE (%)^c^				
< 15	31 (50.8%)	30 (49.2%)	1	1
≥ 15	25 (51.0%)	24 (49.0%)	0.99 (0.47–2.11)	1.00 (0.45–2.21)
Specimen weight (grams)				
< 100	40 (58.8%)	28 (41.2%)	1	1
≥ 100	17 (38.6%)	27 (61.4%)	*2.27 (1.05–4.93)*	2.23 (0.99–5.06)
Axillary clearance				
No	48 (57.8%)	35 (42.2%)	1	1
Yes	13 (36.1%)	23 (63.9%)	*2.43 (1.08–5.44)*	*2.46 (1.09–5.56)*
Neoadjuvant chemotherapy				
No	55 (56.1%)	43 (43.9%)	1	1
Yes	6 (28.6%)	15 (71.4%)	*3.20 (1.15–8.93)*	*3.26 (1.15–9.24)*
Adjuvant chemotherapy				
No	47 (54.0%)	40 (46.0%)	1	1
Yes	14 (43.8%)	18 (56.3%)	1.51 (0.67–3.42)	1.54 (0.67–3.52)
Adjuvant endocrine therapy				
No	15 (48.4%)	16 (51.6%)	1	1
Yes	46 (52.3%)	42 (47.7%)	0.86 (0.38–1.94)	0.82 (0.35–1.89)

*BI-RADS****®*** Breast Imaging Reporting and Data System, *EPBVE* estimated percentage of breast volume excised, *BMI* body mass index

^a^Simple logistic regression model. OR > 1 equals higher likelihood for lower satisfaction (*Q*-score under median value in the BREAST-Q™ BCT domain “Satisfaction with breast”). Presented as fitted model with 95% CI in brackets. Text in italics highlights statistically significant values

^b^Multiple logistic regression model, factor adjusted for age and BMI

^c^Divided at median value of responding patients

**Table 5:** Multiple logistic regression model including factors showing a statistically significant association in the simple logistic regression model

Factor	*Q*-score ≥ 74	*Q*-score < 74	OR (95% CI)^a^	OR (95% CI)^b^
BMI (kg/m^2^)				
< 25	31 (54.4%)	26 (45.6%)	1	1
≥ 25 to < 30	21 (47.7%)	23 (52.3%)	1.28 (0.58–2.84)	1.21 (0.50–2.90)
≥ 30	9 (50.0%)	9 (50.0%)	1.18 (0.40–3.46)	1.08 (0.32–3.68)
Age (years)				
< 50	18 (56.3%)	14 (43.8%)	1	1
50–65	21 (46.7.7%)	24 (53.3%)	1.44 (0.58–3.60)	1.06 (0.39–2.93)
> 65	22 (52.4%)	20 (47.6%)	1.14 (0.45–2.91)	0.81 (0.28–2.33)
BI-RADS® classification of breast density				
High density (C–D)	27 (64.3%)	15 (35.7%)	1	1
Low density (A–B)	34 (44.2%)	43 (55.8%)	*2.32 (1.02–5.29)*	2.00 (0.79–5.06)
Axillary clearance				
No	48 (57.8%)	35 (42.2%)	1	1
Yes	13 (36.1%)	23 (63.9%)	*2.46 (1.09–5.56)*	1.57 (0.52–4.72)
Neoadjuvant chemotherapy				
No	55 (56.1%)	43 (43.9%)	1	1
Yes	6 (28.6%)	15 (71.4%)	*3.26 (1.15–9.24)*	3.02 (0.70–13.12)
Specimen weight (grams)				
≤ 100	40 (58.8%)	28 (41.2%)	1	1
> 100	17 (38.6%)	27 (61.4%)	2.23 (0.99–5.06)	1.45 (0.59–3.54)

*BI-RADS****®*** Breast Imaging Reporting and Data System, *BMI* body mass index

^a^Model where the factor is adjusted for age and BMI. OR > 1 equals higher likelihood for lower satisfaction (Q-score under median value in the BREAST-Q™ BCT domain ‘Satisfaction with breast’). Presented as fitted model with 95% CI in brackets, adjusted for BMI and age. Text in italics highlights statistically significant values

^b^Multiple logistic regression model where all factors shown were adjusted for each other

### Wish for contralateral surgery

Thirteen of the responding women (11%) expressed interest for a contralateral operation. Median time since surgery was 30 months for these women (range 17–53), and in most cases, they had previously not expressed a wish (*n* = 5) for contralateral surgery at clinical follow-up. In some cases (*n* = 4), the patients had too high BMI to be subjected to a contralateral reduction mammaplasty according to the National Guidelines. In two cases, there was an indication for contralateral surgery according to both the surgeon and patient; however, a decision to further await contralateral surgery was made, since the effects of postoperative radiotherapy and/or endocrine therapy were expected to further affect the breast. One patient had the clinical follow-up at the oncological department and had therefore not been in contact with the surgical department. One patient was on the waiting list for a contralateral procedure.

The women who wished to have contralateral surgery for symmetry reported a higher frequency of dissatisfaction regarding the size and shape of the operated breast and with postoperative symmetry between the breasts (Table 6).

**Table 6:** Satisfaction with different aspects of the operated breast for patients who wished to have contralateral surgery for symmetry

Would you like to have a procedure of the contralateral breast for symmetry?^a^					
Satisfaction with aspect of the operated breast^a^	Yes (*n* = 13)		No (*n* = 101)		*p* value
	Satisfied^b^	Dissatisfied	Satisfied	Dissatisfied	
Appearance of the operated breast	10 (76.9%)	3 (23.1%)	92 (91.1%)	9 (8.9%)	0.139
Size of the operated breast	8 (66.7%)	4 (33.3%)	92 (91.1%)	9 (8.9%)	0.032*
Shape of the operated breast	8 (66.7%)	4 (33.3%)	94 (93.1%)	7 (6.9%)	0.017*
NAC positioning	10 (83.3%)	2 (16.7%)	94 (93.1%)	7 (6.9%)	0.224
NAC appearance	8 (72.7%)	3 (27.3%)	90 (89.1%)	11 (10.9%)	0.140
Symmetry	4 (33.3%)	8 (66.7%)	86 (85.1%)	15 (14.9%)	< 0.001*
Appearance of scar	9 (69.2%)	4 (30.8%)	86 (86.9%)	13 (13.1%)	0.109
Skin sensitivity	7 (58.3%)	5 (41.7%)	82 (82.0%)	18 (18.0%)	0.068

Descriptive statistics of dichotomized answers from the study-specific questionnaire, sub grouped into women who wished to have contralateral surgery compared to those who did not. Presented as count, percentage of sup group in brackets

Fishers exact test was used to find differences between patients who wished to have contralateral surgery for symmetry and those who did not. *p* value < 0.05 is considered to represent a significant difference between groups

^a^No missing values were included

^b^Satisfied defined as score of “satisfied” or “very satisfied” and dissatisfied defined as a score of “not entirely satisfied” or “dissatisfied” for the aspect of the operated breast evaluated

### Correlation between “Satisfaction with breasts” and other BREAST-Q™ BCT domains

The domain “Satisfaction with breasts” had positive correlation coefficients with all other BREAST-Q™ BCT domains. “Sexual well-being” was found to have the highest correlation (Spearman’s rho = 0.584, moderate) and “Psychosocial well-being” the second highest correlation (rho = 0.562, moderate). Correlations are presented in Table 7 along with *p* values.

**Table 7:** Correlation between “Satisfaction with breast” and other BREAST-Q™ BCT domains

	Other BREAST-Q™ BCT domains		
	Correlation	Interpretation^a^	*p* value
1. Adverse effects of radiation	0.186	Very weak	0.043*
2. Psychosocial well-being	0.562	Moderate	< 0.001*
3. Sexual well-being	0.584	Moderate	< 0.001*
4. Physical well-being	0.389	Weak	< 0.001*
5. Satisfaction with information from breast surgeon	0.441	Moderate	< 0.001*
6. Satisfaction with breast surgeon	0.214	Weak	0.020*
7. Satisfaction with medical team	0.228	Weak	0.013*
8. Satisfaction with office staff	0.182	Very weak	0.051

Correlation analysis performed using Spearman’s correlation assessing rank correlation between BREAST-Q™ BCT domain 1 “Satisfaction with breasts” and remaining domains

^a^Spearman’s correlation coefficient (rho) measures the strength of the monotonic relationship between paired data and may lie between − 1 and 1. 0–0.19 = very weak, 0.2–0.39 = weak, 0.4–0.59 = moderate, 0.6–0.79 = strong, 0.8–1 = very strong. *p < 0.05 is considered a statistically significant correlation

## Discussion

During the last decade, the use of oncoplastic surgery has increased rapidly. Intentions have been to widen the implementation of BCT, thus avoiding mastectomy or increasing patient satisfaction in challenging cases [13, 20]. The BREAST-Q™ questionnaires are currently the only available validated and disease-specific PROMs for subjective auto-evaluation after breast cancer surgery. To our knowledge, there are to date five published studies that have used BREAST-Q™ BCT, two of them evaluating OPS [23, 27] and the other three evaluating non-specified BCS [24–26]. Out of these studies, Dahlbäck et al. [24] and O’Connell et al. [25] are considered appropriate for comparison since they presented median values and had the highest response rates (Table 8).

**Table 8:** Previous studies evaluating patient satisfaction/QoL with the BREAST-Q™ BCT compared to the current study

	First author of study		
	Dahlbäck*	O’Connell**	Current study
Year published	2017	2016	
Evaluated surgical method(s)	BCT/WLE	BCT/WLE	OPS
Time period when operations were performed	2008–2012	2009–2015	2013–2016
Response rate	71%	58%	83%
Number of responding participants	348	200	120
Radiological tumor extent (mm)	15 (median value)	16 (mean value)	20 (median value)
Weight of specimen excised (grams)	–	32.5 (mean value)	86 (median value)
Breast Q™ BCT domains			
“Satisfaction with breasts”	66 (57–80)^a^	68 (55–80)	74 (59–85)
“Effects of radiotherapy”	100 (89–100)	89 (78.25–100)	100 (89–100)
“Psychosocial well-being”	82 (61–100)	82 (63–100)	87 (57–100)
“Sexual well-being”	60 (48–79)	57 (45–66)	60 (49–69)
“Physical well-being”	81 (69–92)	75 (64–86)	78 (69–92)
“Satisfaction with information from breast surgeon”	62 (53–84)	77 (64–100)	75 (58–100)
“Satisfaction with breast surgeon”	100 (81–100)	100 (100–100)	100 (92–100)
“Satisfaction with medical team”	100 (92–100)	100 (100–100)	100 (100–100)
“Satisfaction with office staff”	100 (93–100)	100 (100–100)	100 (100–100)

Summary of the three studies using BREAST-Q™ BCT that have presented median or mean values for one or several domains

*BCT* breast-conserving therapy, *OPS* oncoplastic surgery, *WLE* wide local excision

^a^Values are median and interquartile range in brackets

*Dahlback et al. 2017 [26]

**O’Connell et al. 2016 [27]

Even though tumor extent and resected specimen weight were larger in the current study, the median *Q*-score of the BREAST-Q™ domain “Satisfaction with breasts” was higher than the *Q*-scores presented by O’Connell and Dahlbäck et al. [26, 27]. A slightly higher median *Q*-score was also found in the domain “Psychosocial well-being,” and it was also slightly higher or equal in the domain “Sexual well-being” in the current study compared to the other studies. These three domains are considered most likely to be affected by a poor esthetic outcome, supported by the fact that the domains “Psychosocial well-being” and “Sexual well-being” showed the highest rate of correlation with the domain “Satisfaction with breast” in our study. “Satisfaction with breast” correlated poorly with how participants perceived their contacts with medical care professionals, suggesting that patients are able to dissociate the different aspects of their treatment when responding to the questionnaire. Similar correlation coefficients were found by O’Connell et al. In similarity to both other studies, the domain “Sexual well-being” had a lower response rate than the other domains [26, 27]. One reason might be that many women are no longer sexually active, as they chose not to answer questions regarding “Sexual well-being” by marking “not applicable.” A difference between the comparable study cohorts, apart from surgical technique (OPS in the current study compared to different and mixed methods of BCT), is that patients in the other two studies were treated during time periods prior to the current study (O’Connell et al. 2009–2015, Dahlbäck et al. 2008–2012). Since the use of OPS has increased during the latest years, perhaps some patients in these other cohorts should have received OPS, if current recommendations had been present then.

In the current study cohort, many previously studied potential risk factors for lower patient satisfaction after BCT did not significantly affect the OR of having a *Q*-score of the BREAST-Q™ BCT domain “Satisfaction with breast” below the median value. Regarding the impact of EPBVE, women that had a resection of > 15% of the breast were as likely to be satisfied as the women where < 15% of the breast had been removed. This indicates that a high EPBVE not necessarily impairs the esthetic outcome if techniques of OPS are used and that OPS can thus be beneficial when large resections are necessary. Axillary clearance, neoadjuvant chemotherapy, resected weight > 100 g, and low breast density were associated with lower patient satisfaction in the univariate analysis, and all but specimen weight remained statistically significant when adjusted for age and BMI. However, when tested in a multiple logistic regression model, no independent risk factors were found. One reason for this may be that many of the factors covariate. For example, all but one patient who received neoadjuvant chemotherapy were also operated in the axilla, and patients with low-density breasts tended to have larger breasts, why they were more likely have had resections of > 100 g. It is thereby difficult to differentiate the true impact of the factors studied, since the number of study individuals is too low to make representative subgroups. In a larger study group, significant values would more likely have been found. Further studies evaluating risk factors for lower patient satisfaction and negative effects on HR-QoL after OPS are therefore suggested.

A majority of the women treated with oncoplastic volume displacement techniques did not express a wish for a contralateral procedure for symmetry. This finding supports the approach to offer contralateral surgery in the primary setting only in selected cases, and otherwise as a secondary procedure after individual assessment. Since PRT can cause unpredictable changes to the breast [17, 18], it might be better to wait until the 1-year follow-up visit to see if contralateral surgery is needed. Achieving a better long-term symmetrical appearance when the “template,” in form of the cancer-treated breast, has reached its final outcome could be easier than trying to predict the change over time and compensate for this in the primary setting. Bilateral surgery also demands higher health care costs due to longer operating time and/or more surgeons. In Sweden, as in other countries with a tax-financed healthcare system, the awareness of costs and benefits for healthcare procedures has to be especially high. In addition, effects of contralateral surgery, such as influencing future detection of an eventual later contralateral cancer negatively [38], scars and potential loss of sensation to the areola and increased risk of peri- and postoperative complications [39], must also be taken into consideration.

The strengths of the study include the use of a validated questionnaire, which limits the researcher’s imposition. In addition, efforts to remove confounders were made. All patients received PRT, which is a well-known factor that could compromise esthetic outcome. Also, all patients had a contralateral untouched breast, meaning that they could compare the operated breast to an untouched one. This enabled them to be their own controls, to see if the goal of preserving the natural shape and size of the breast was achieved. Another strength in this study was the high response rate. As for most questionnaire-based studies, selection bias cannot be completely ruled out. Perhaps very dissatisfied and very satisfied patients are more prone to respond than patients in the middle of the spectrum, giving more extreme results for the responders compared to the total cohort. Further limitations of the study include the lack of a control group with patients operated with other methods of BCT. The results of this study were therefore compared to groups of women in other studies, with different methods of surgery and postoperative treatment. The current cohort did not include patients operated with volume reduction mammoplasty, i.e., patients selected for this type of oncoplastic procedure were excluded from the material. Since these operations usually are performed bilaterally, perhaps some patients who wanted bilateral surgery from the start were enrolled to volume reduction surgery instead of volume displacement surgery for that reason. In addition, six patients subjected to oncoplastic displacement surgery had been operated with bilateral surgery to achieve symmetry. These patients could not be included in the current study, since the objective was for the patient to compare the operated breast to an untouched one. With only six patients, it would have been difficult to draw any reliable conclusions, why no further analyses were attempted for this group in this material. However, this poses a risk of selection bias in the current cohort, since these patients in fact were not satisfied with unilateral surgery. Also, due to the retrospective design of this study, only the postoperative BREAST-Q™ BCT module was used. Since the BREAST-Q™ BCT has a preoperative module, it would have been valuable to create a baseline measurement, to illustrate the change of well-being pre- and postoperatively. Especially in the psychosocial and sexual domains, in which a change in the patients view of herself, psychological health or sexuality are aspects of interest.

In this study, the patients’ level of satisfaction and aspects of HR-QoL were the primary end-points. As these results are subjective, it could be valuable to evaluate the esthetic result also by more objective evaluation modalities, such as software-based methods (for example BCCT.core [40, 41]), in future studies.

## Conclusion

In this study cohort of breast cancer patients, treated unilaterally with oncoplastic volume displacement surgery and with an untouched contralateral breast, the median *Q*-score of the BREAST-Q™ domain “Satisfaction with breast” was slightly higher than those presented in other previously published studies evaluating BCS with BREAST-Q™, despite larger resections. The results indicate that oncoplastic volume displacement techniques can be beneficial in the surgical treatment of breast cancer for selected patients. In this study, no independent risk factor for lower patient satisfaction was identified. Larger study cohorts are needed to further investigate potential risk factors for lower patient satisfaction after oncoplastic surgery. Most patients in the current study were not interested in a contralateral procedure. In patients treated with oncoplastic volume displacement surgery, contralateral surgery for symmetry is suggested to be performed only after individual evaluation and as a delayed procedure.

## Data Availability

The datasets generated and/or analyzed during the current study are not publicly available due to the integrity of the individual participants in the study but are available from the corresponding author on reasonable request.

## Abbreviations

BCS: Breast-conserving surgery
BCT: Breast-conserving therapy
BMI: Body mass index
CI: Confidence interval
EPBVE: Estimated percentage of breast volume excised
HR-QoL: Health-related quality of life
IQR: Interquartile range
NAC: Nipple-areolar complex
NCSP: NOMESCO classification of surgical procedures
OPS: Oncoplastic breast-conserving surgery
OR: Odds ratio
PROM: Patient-reported outcome measure
PRT: Postoperative radiotherapy
WLE: Wide local excision
